# Current use of antithymoglobulin as induction regimen in kidney transplantation: A review

**DOI:** 10.1097/MD.0000000000037242

**Published:** 2024-03-01

**Authors:** Byung Hwa Park, Ye Na Kim, Ho Sik Shin, Yeonsoon Jung, Hark Rim

**Affiliations:** a Renal Division, Department of Internal Medicine, Gospel Hospital, Kosin University College of Medicine, Busan, South Korea; b Transplantation Research Institute, Kosin University College of Medicine, Busan, South Korea.

**Keywords:** antithymoglobulin, immunosuppressant, induction, kidney transplantation

## Abstract

Currently, various immunosuppressive drugs are used in organ transplantation. In particular, antithymoglobulin is a widely used drug in kidney transplantation in Korea, accounting for 20% of all induction therapy. According to existing studies, antithymoglobulin induction therapy has several advantages and disadvantages compared with other immunotherapies depending on the kidney transplant situation (dead donor, living donor, low-risk recipient, and high-risk recipient) or antithymoglobulin dose. In this review, we summarize the research conducted so far on antithymoglobulin and hope that antithymoglobulin research on kidney transplantation will be actively conducted in the future.

## 1. Introduction

Over the past half century, advances in surgical techniques and immunology have increased the success rate of organ transplantation. With these advances, organ transplant research is now focused on improving patient survival and quality of life and making transplants available to more patients. In particular, various immunosuppressants are used in transplantation, and continued research on immunosuppressants is necessary for successful organ transplantation in the future.^[1]^

**Figure 1.**
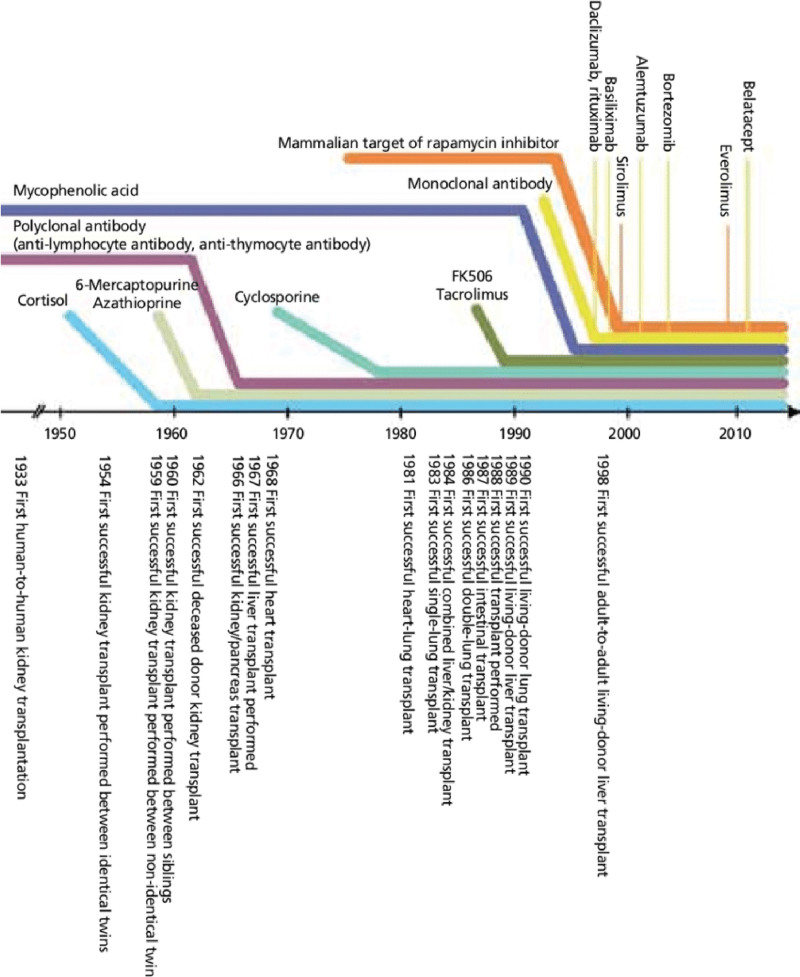
Progress of organ transplantation and history of immunosuppressant use for clinical transplantation.

Immunosuppression methods in kidney transplantation are divided into initial induction immunosuppression therapy and long-term maintenance immunosuppression therapy. Among them, induced immunosuppression therapy is a method of inducing strong immunosuppression in the early stages of transplantation to reduce acute rejection and delayed graft function and reduce the occurrence^[1]^ of nephrotoxicity caused by calcineurin inhibitor (CNI) (Fig. 1). It is mainly used in combination with antibodies against lymphocytes and general immunosuppressants, of which antithymoglobulin is commonly used. In Korea, the use of antithymoglobulin as induction therapy accounts for 20% of all kidney transplantation, making antithymoglobulin an important drug in kidney transplantation. In Korea, due to the short travel distance, the use of antithymoglobulin is less than that in other countries, currently accounting for 20% of all kidney transplants. However, as the frequency of use is increasing, antithymoglobulin has become an important drug in kidney transplantation.^[1]^

Antithymoglobulin is a purified gamma globulin used to treat human thymocytes in horses and rabbits. In kidney transplantation, antithymoglobulin immunosuppressants attach to the T-cell surface during induction, blocking stimulation from entering the T cell and inducing antibody-dependent cell-mediated cytotoxicity, leading to T-cell apoptosis. Additionally, it induces immune reconstitution through immune regulation by generating specific agonists, especially CD4^+^CD25^+^Foxp3^+^ regulatory T cells. Due to this principle, antithymoglobulin is currently in the spotlight as a treatment for acute rejection that does not respond to induction therapy or steroids after transplantation in organ transplantation.^[1]^

Immunosuppressants can be divided into T cell–mediated rejection immunosuppressants and antibody-mediated rejection immunosuppressants. Among T cell–mediated rejection immunosuppressants, antithymoglobulin is a polyclonal antibody against T lymphocytes and purified gamma globulin used to treat human thymocytes in horses and rabbits.^[2]^ In renal transplantation, antithymoglobulin immunosuppressive agents attach to the surface of T cells during induction, block stimuli from entering T cells, and induce antibody-dependent cell-mediated cytotoxicity to induce T-cell apoptosis. In addition, it induces immune reconstitution through immunomodulation, especially by generating specific actions, such as CD4^+^CD25^+^Foxp3^+^ regulatory T cells. Owing to this principle, antithymoglobulin in organ transplantation is currently highlighted as a treatment for acute rejection that does not respond to posttransplant induction therapy or steroids.^[3]^

Through this review, we will summarize the research on antithymoglobulin that has been conducted to date, recall its importance, and find out directions for more effective use of antithymoglobulin research in kidney transplantation that may take place in the future.

## 2. U.S. organ procurement and transplantation data indicates several advantages and disadvantages were identified compared with other drugs when using antithymoglobulin as induction therapy

According to the U.S. Organ Procurement and Transplantation Data, induction therapy is a commonly used treatment in kidney transplantation, and the acute rejection that occurred in 2019 was investigated as interleukin-2 reactive antibody (8.4 %), antilymphocyte antibody (6.9 %), and no induction (6.6 %). In addition, as a result of using r-antithymoglobulin (rATG) compared with e-antithymoglobulin (eATG), the incidence of biopsy-proven acute rejection (BPAR) was reduced, allograft/patient survival improved, and the incidence of cytomegalovirus infection reduced. Leukopenia and posttransplant lymphoproliferative disorders (PTLD) have high incidences. (Posttransplant lymphoproliferative disorders are affected by the type and dose of immunosuppression regimens and cytomegalovirus infection particularly r-antithymoglobulin has been reported to increase the risk of posttransplant lymphoproliferative disorders in kidney transplantation.)^[4,5]^ Compared with basiliximab, r-antithymoglobulin reduced the incidence of biopsy-proven acute rejection and cytomegalovirus infection. The incidence of leukopenia has been extensively investigated.^[6]^

## 3. Antithymoglobulin induction therapy is more effective in acute rejection and long-term prognosis than Basiliximab

A prospective randomized international study was conducted after administering antithymoglobulin 1.5 mg/kg/day and basiliximab (20 mg) to high-risk (Table 1)^[7]^ kidney transplantation recipients who underwent deceased donor kidney transplantation. As a result, the incidence of acute-treated biopsy-proven antibody rejection was low in the antithymoglobulin treated patient group. In addition, the cytomegalovirus infection incidence was lower in the antithymoglobulin administered patient group. In other words, in patients with an acute rejection risk, 5 days of antithymoglobulin induction therapy could reduce the incidence and severity of acute rejection; however, the delayed graft failure and patient and graft survival rates were similar. Even after 5 years of long-term follow-up, the effect of antithymoglobulin induction therapy was sustained and stable compared with basiliximab, and this strategy was shown to be cost-efficient in terms of long-term efficacy and patient safety.^[8]^

**Table 1:** Overview of pretransplant risk factors for acute rejection after kidney transplant.

Risk factor	Importance	Comment
Recipient age Younger age Adolescence	++ +++	Stronger immune response Higher risk for nonadherence
Donor age	+	Higher immunogenicity in older organs
Recipient gender	+	Fewer rejections in males
Ethnicity	+++	Significantly higher risk of rejection in African Americans
Deceased vs living donor	+	Few differences (also little difference between deceased donation after cardiac vs brain death, or expanded vs criteria donation)
Previous-transfusion	+	Considered “low immunologic responder” if patient is unsensitized despite previous transfusion
Previous-transplantation	++^*^	No relevant increase in risk if the patient remains unsensitized despite prior transplantation. Early loss of previous graft to immunological causes increases risk of rejection after next graft
Previous-pregnancy	++	Increasing risk with successive pregnancies
PRA > 0% (HLA antibodies)	+++	Applies to both historic and current PRA level, HLA antibodies class I and/or class II
Preformed HLA, DSA (>500 MFI)	++++	Having no preformed HLA DSA at transplant is associated with low immunological risk; low levels of noncytotoxic HLA antibodies confer intermediate risk. De novo HLA DSA posttransplant monitoring is required
AT1 receptor antibodies	++	Test is relatively widely available
T-cell ELISPOT	++	Time-consuming (1–2 d) and requires large blood volume; may be more relevant for living-donor transplants
Soluble CD30	+	Inconclusive data
Sensitized patients after desensitization	++/+++	Increased risk of AMR appears to be sustained after desensitization in DSA-positive patients with negative cytotoxicity and flow cross-match, but to a far lesser extent than in patients with positive cytotoxic (profound increase in risk) or flow cross-match (moderate increase in risk)
HLA mismatch	+++	Marked and well-documented effect on cellular and antibody-mediated rejection. Particularly pronounced for HLA DR mismatch
CMV mismatch	–	No association between CMV mismatch and acute rejection due to CMV prophylaxis
EBV mismatch	–	No effect per se on acute rejection
Cold ischemia time	+	Less important with current shorter ischemic times
Machine preservation	+	Minor effect versus cold storage; not well-documented
Delayed graft function	+++	Delayed function may prompt changes to the planned protocol in the first few days posttransplant

AMR = antibody mediated rejection, AT1 = angiotensin II receptor type 1, CMV = cytoemgalovirus, DSA = donor-specific antibodies, EBV = Epstein-Barr virus, HLA = human leucocyte antigen, MFI = mean fluorescence intensity, PRA = panel reactive antibody.

## 4. Low-dose antithymoglobulin could be an effective induction therapy in deceased donor kidney transplantation, low-immunological risk patient

When performing deceased donor kidney transplantation in patients with low immunological risk, high-dose antithymoglobulin, low-dose antithymoglobulin, and basiliximab were administered. In this retrospective, single center study, the patients in the low-dose antithymoglobulin group were older (*P* < .001) and had higher serum creatinine levels (*P* < .001). Regarding graft failure, the low-dose antithymoglobulin group was not significantly different from the basiliximab group (*P* = .080); however, a significant difference (*P* = .004) was observed in the antithymoglobulin dose group. The cytomegalovirus infection rate was significantly lower with basiliximab, but no significant differences were found in the rates of polyoma (BK) virus, bacterial, or fungal infections. In summary, when low-dose antithymoglobulin therapy was administered, significant differences were found in graft survival and patient survival rates compared with basiliximab therapy, even though the donor was older, or the serum creatinine level was higher. Therefore, this study concluded that low-dose antithymoglobulin could be an effective induction therapy.^[9]^

## 5. Low-dose antithymoglobulin as induction therapy had better outcomes than Basiliximab in low-risk living donor kidney transplantation

This retrospective, single-center study compared graft survival and patient survival between a low dose antithymoglobulin administered group and a basiliximab administered group in low-risk living donor kidney transplantation. Better results were obtained for rejection, graft function, and graft survival in the low-dose antithymoglobulin administered group than in the basiliximab group. Although cytomegalovirus infection and polyoma (BK) viral infections were more frequent in the antithymoglobulin treated group, the results showed that treatment was possible through early detection and management.^[10]^

## 6. There was no difference in kidney transplantation prognosis of antithymoglobulin and Basiliximab in early rapid steroid withdrawal

Steroids are used as immunosuppressants in kidney transplantation. Steroid-related complications include hypertension, posttransplant diabetes mellitus, peripheral fractures, avascular necrosis, and cataracts.^[6,11]^

In a study comparing acute rejection and new-onset diabetes mellitus after transplantation between steroid avoidance and steroid maintenance groups, it was confirmed that the steroid avoidance group had an increased risk of acute rejection but reduced posttransplant diabetes and death.^[12]^

In a comparative study of r-antithymoglobulin and basiliximab induction conducted for rapid steroid withdrawal after renal transplantation, the primary endpoint, biopsy-proven antibody rejection was similar at 12 months. In the secondary endpoint and infection, there was no significant difference between basiliximab + steroid, basiliximab + rapid steroid withdrawal (RSWD), and antithymoglobulin + rapid steroid withdrawal. Taken together, antithymoglobulin did not show superiority in preventing biopsy-proven antibody rejection compared with basiliximab when rapid steroid withdrawal was performed for 1 year kidney posttransplantation.

Nevertheless, rapid steroid withdrawal after induction therapy in patients with a low immunological risk profile can be achieved without a loss of efficacy and is advantageous with regard to the incidence of posttransplantation diabetes.^[13]^

In a randomized controlled trial of steroid avoidance in immunologically low-risk kidney transplant recipients, further evidence of the feasibility, safety, and efficacy of early steroid-free treatment was obtained in the first 2 years posttransplantation. Although a significant reduction in the incidence of posttransplantation diabetes mellitus was not observed with the steroid-avoidance regimen in this selected group at low risk for diabetes, it may be a preferred treatment option in recipients who are deemed to be at high risk for posttransplantation diabetes mellitus or have multiple comorbidities.^[14]^

## 7. Antithymoglobulin induction therapy with early rapid steroid withdrawal therapy in renal transplantation resulted in higher allograft survival rate than antiinterleukin 2R therapy

In this study, antithymoglobulin induction therapy was associated with fewer rejection episodes and antithymoglobulin induction therapy is associated with higher patient and allograft survival rates than antiinterleukin 2R therapy.^[15]^

## 8. In long-term follow-up at high immunological risk showed after 5 years, survival of deceased donor grafts was significantly better in low-dose antithymoglobulin than Basiliximab

When investigating the long-term safety and efficacy of antithymoglobulin induction using integrated national registry data to achieve ten-year follow-up of 10-10. Study participants in high immunological risk patients, no significant difference was found between ATG and basiliximab administration.^[16]^

When the 8-year follow-up of low-dose antithymoglobulin and basiliximab induction therapy was performed in patients with low immunological risk, rejection was reduced in the low-dose antithymoglobulin administered group compared with the basiliximab administered group, and the 5-year patient survival and 5-year graft survival showed significantly better results. When comparing patient survival over 8 years, there was no significant difference. At 5 years, deceased donor graft survival was significantly better in the low-dose antithymoglobulin -treated recipients than in the basiliximab-treated group.^[17]^

In conclusion, in Korea Data-High-risk recipients, when comparing the optimal dose of thymoglobulin for induction therapy, there were no differences in graft survival, infectious disease, or hematological problems between the 2 groups.

Low-dose antithymoglobulin was as effective as high-dose antithymoglobulin in preventing acute rejection and delayed graft failure in high-risk patients. The incidence of complications, such as infection and hematologic problems, did not differ between the 2 groups. Although further studies are needed to lower the dose of antithymoglobulin, this study suggests that less than 6 mg/kg antithymoglobulin is sufficient for the prevention of acute rejection and delayed graft failure in high-risk patients.^[18]^

## 9. There was no difference in the results of low-dose antithymoglobulin and standard-dose antithymoglobulin as an induction therapy undergoing early steroid withdrawal

In this study, low-dose antithymoglobulin (2.25 mg/kg) was a promising option as an induction therapy in low-risk patients with no significant differences in acute rejection or graft function when compared with standard-dose antithymoglobulin (3.75 mg/kg), and with possibly fewer leukopenia and infectious complications. In addition, peripheral blood cell analyses showed similar T-cell depletion efficiency with a lower dose of antithymoglobulin.^[19]^

## 10. Antithymoglobulin and intravenous immunoglobulin in patients had higher graft survival rate and lower rejection rate compared with the control group in low-level donor-specific human leucocyte antigen antibodies

In this study, the cumulative incidence of clinical/subclinical antibody-mediated rejection within 6 months posttransplant was lower in the antithymoglobulin/intravenous immunoglobulin group than in the control group. Clinical antibody-mediated rejection and T cell-mediated rejection were significantly lower in the antithymoglobulin/intravenous immunoglobulin group than in the control group. The patient and graft survival rates did not differ between the 2 groups; however, this result must be interpreted with caution because the follow-up time in the antithymoglobulin/intravenous immunoglobulin group was very short. The absence of antithymoglobulin/intravenous immunoglobulin induction therapy was the only independent predictor of clinical antibody-mediated rejection.^[20]^

However, absence of antithymoglobulin/intravenous immunoglobulin induction therapy was the only independent predictor of antibody-mediated rejection. Therefore, antithymoglobulin/intravenous immunoglobulin induction significantly reduced the incidence of T cell-mediated rejection and severity of antibody-mediated rejection in patients with low-level human leukocyte antigen-donor specific antibody. In a clinical study to evaluate the utility of induction therapy in kidney transplantation recipients at low immunological risk, it reduced the incidence of T-cell and antibody-mediated rejection regardless of donor and recipient human leukocyte antigen antibodies matching.^[21,22]^

In another observational clinical/subclinical antibody-mediated rejection rate of 38% suggests insufficient control of the humoral immune response. Adaptation/supplementation of the regimen with drugs that might target the humoral immune response more specifically could be beneficial but needs to be studied in prospective randomized trials.^[20]^

## 11. Low-dose antithymoglobulin as an induction therapy showed no acute rejection in living donor renal transplants with a negative cross-match and pretransplant donor-specific antibody

In this study, when a lower dose of antithymoglobulin (1.5 mg/kg) was administered as a 3-day course, acute rejection was not observed in the donor specific antibody positive group and was confirmed in one event in the donor specific antibody negative group, which was significant.^[23]^

## 12. In kidney transplantation using a donation after brain death donor with acute kidney injury, low-dose antithymoglobulin resulted in better graft survival than Basiliximab

In conclusion, acute kidney injury in donors after brain death negatively affects the delayed graft failure rate. However, it did not affect the long-term graft function or death-censored graft survival. Low-dose antithymoglobulin may be considered for immunosuppressive induction in kidney recipients with acute kidney injury because it produces better graft survival than basiliximab.^[24]^

## 13. In kidney transplantation in elderly patients within the Korean multicentric registry, antithymoglobulin was more effective than Basiliximab in terms of prognosis and reduction of complications

In this study, compared with basiliximab, antithymoglobulin reduced tacrolimus and steroid requirements without differences in all-cause mortality, rejection, or infection in elderly low-risk kidney transplant patients, resulting in a reduced incidence of new-onset diabetes mellitus after transplantation incidence.^[25]^

## 14. Low-dose antithymoglobulin had lower risk of subclinical and clinical rejection than standard-dose antithymoglobulin in kidney transplantation

Patients were divided into low-dose antithymoglobulin patients who received less than 5 mg/kg antithymoglobulin and regular-dose antithymoglobulin patients who received higher than 5 mg/kg antithymoglobulin, and the occurrence of rejection was investigated; antithymoglobulin doses lower than 5 mg/kg may be associated with a heightened risk of rejection despite a low degree of sensitization.^[26]^

## 15. Conclusion

This review, we summarized how antithymoglobulin has been used and studied to date in kidney transplantation and the antithymoglobulin research conducted so far is listed in Table 2.

**Table 2:** Rabbit antithymoglobulin induction therapy in kidney transplant.

	BPAR	DGF	Allograft survival	Patient survival	CMV	Other
Basiliximab vs rATG (high-risk DDKT)	B > A	B = A or B < A	B = A	B = A	B > A	Myelosuppression: B < A
Basiliximab vs low-dose rATG vs high-dose rATG (low-risk DDKT)		B < LD A	B = LD A B < HD A	B = LD A = HD A	B < LD A	BK viremia: B = LD A = HD A
Basiliximab vs low dose rATG (low-risk LDKT)	B = LD A	B = LD A	B = LD A	B = LD A	B < LD A	De Novo DSA: B < LD A BK viremia: B < LD A
Basiliximab + steroid vs basiliximab + RSWD vs rATG + RSWD (low risk KT)	similar		similar	similar	similar	BK viremia: Similar PTDM risk↓in RSWD
Basiliximab + steroid vs rATG + steroid avoidance (low risk KT)	Similar		similar	similar		PTDM risk similar in 2 groups
Basiliximab + ESW vs rATG + ESW (LDKT, DDKT)	B > A		B < A	B < A		PTDM: B > A
Basiliximab vs rATG (high risk KT, 10 y)	B = A			B = A		Freedom from acute rejection, graft failure or death: B < A (until 1.5 y)
Basiliximab vs rATG (low-risk LDKT/DDKT, 8 y)	B > A in LD		B < A in DD	B < A in DD		
Low dose rATG vs high dose rATG (high risk KT)	similar	similar	similar			
Standard- dose rATG + ESW vs Low dose rATG + ESW (low risk KT)	similar	similar			similar	BK viremia: similar
rATG + IVig vs No (low level of DSA)	A + IVig < No		similar	similar		
DSA (+) vs DSA (-) (high risk LD	similar					
AKI vs No AKI (basiliximab, high dose ATG, low dose ATG in DDKT)		AKI > No	LD A > B LD A > HD A			
Basiliximab vs rATG (Age > 60, low risk KT)	similar		similar	similar		PTDM: B > A
Low dose rATG vs regular dose rATG (low and high risk KT)	LD A > HD A					

ATG = antithymoglobulin, B = Basiliximab, BPAR = biopsy-proven acute rejection, CMV = cytomegalovirus, DDKT = deceased donor kidney transplantation, DGF = delayed graft failure, DSA = donor-specific antibodies, ESW = early steroid withdrawal, KT = kidney transplantation, LDKT = living donor kidney transplantation, rATG, r-antithymoglobulin, RSWD = rapid steroid withdrawal.

Overall, antithymoglobulin induction is used to treat vascular, steroid resistance, and antibody-mediated rejection.

According to the KDIGO guidelines, it is recommended to be used to prevent and treat acute rejection, especially in patients with high immunological risk.^[27,28]^

However, antithymoglobulin has several side effects, such as cytokine release syndrome (anaphylaxis, fever, chills, dyspnea, vomiting, hypotension, and rash) and delayed reactions (serum disease, infection). In addition, several side effects have been confirmed, such as a 75% increase in malignancy and a 32% increase in cytomegalovirus disease, so this drug should be used in a planned monitoring environment when administered to patients. This may be because data for using antithymoglobulin have not yet been released to clearly determine the optimal dosing regimen, and the benefit-risk balance of antithymoglobulin administration must be understood.^[29]^

In the future, large-scale multicentric prospective studies of antithymoglobulin will be needed to reduce side effects.

These results will serve as an important foundation for increasing the success rate of kidney transplantation in the future, providing kidney transplant opportunities to more patients and reducing side effects.^[30,31]^

## Author contributions

**Conceptualization:** Ho Sik Shin.

**Formal analysis:** Ho Sik Shin.

**Investigation:** Ho Sik Shin.

**Methodology:** Ho Sik Shin.

**Supervision:** Ho Sik Shin, Yeonsoon Jung, Hark Rim.

**Validation:** Byung Hwa Park, Ho Sik Shin, Yeonsoon Jung, Hark Rim

**Writing—original draft:** Byung Hwa Park.

**Writing—review & editing:** Byung Hwa Park, Ye Na Kim, Ho Sik Shin.
